# IL-1 family cytokines and soluble receptors in systemic lupus erythematosus

**DOI:** 10.1186/s13075-018-1525-z

**Published:** 2018-02-08

**Authors:** Paola Italiani, Maria Laura Manca, Francesca Angelotti, Daniela Melillo, Federico Pratesi, Ilaria Puxeddu, Diana Boraschi, Paola Migliorini

**Affiliations:** 1 Institute of Protein Biochemistry, National Research Council, Naples, Italy; 2 Clinical Immunology Unit, Department of Clinical and Experimental Medicine, University of Pisa, Pisa, Italy

**Keywords:** Il-1 family, Systemic lupus erythematosus, Biomarkers, Soluble IL-1 family receptors

## Abstract

**Background:**

Dysregulated production of cytokines has a critical role in systemic lupus. The aim of this work is to identify, by a comprehensive analysis of IL-1 family cytokines and receptors in serum, correlation between cytokines/receptors’ levels and the clinical and serological features of the disease.

**Methods:**

A full clinical evaluation was performed in 74 patients with systemic lupus erythematosus (SLE). C3, C4, anti-dsDNA and anti-C1q antibodies were measured. Cytokines of the IL-1 family (IL-1α, IL-1β, IL-33, IL-18), soluble receptors (sIL-1R1, sIL-1R2, sIL-1R3, ST2/sIL-1R4) and antagonists (IL-1Ra, IL-18 binding protein (IL-18BP)) were measured in serum by multiarray ELISA. Free IL-18 was calculated as the amount of IL-18 not inhibited by IL-18BP. Data were analysed by non-parametric tests and by multivariate analysis, using partial least squares (PLS) models.

**Results:**

Total IL-18, IL-18BP, sIL-1R4 and IL-1Ra levels were higher in SLE vs. controls. Total and free IL-18 and sIL-1R4 were higher in patients with active vs. inactive disease and correlated with ECLAM, anti-C1q and anti-dsDNA antibodies. sIL-1R2 was higher in patients with inactive disease, was negatively correlated with ECLAM and anti-C1q antibodies and was positively correlated with C3 levels. PLS identified sIL-1R4, sIL-1R2 and anti-dsDNA as variables distinguishing patients with active from those with inactive disease; sIL-1R4, IL-18BP and anti-dsDNA identified patients with active nephritis; sIL-1R4, C3, IL-18 and free IL-18 identified patients with haematological involvement.

**Conclusion:**

The data support the use of IL-18, sIL-1R2 and sIL-1R4 as biomarkers of disease activity and organ involvement, and suggest that failure in the inhibition of IL-1 activation may be a critical event in the active stages of SLE.

## Background

Systemic lupus erythematosus (SLE) is an autoimmune disease characterised by the production of a variety of autoantibodies directed against highly conserved molecules, mainly but not exclusively localised in the cell nucleus.

Dysregulated production of a number of cytokines, including those of the IL-1 family, has a critical role in the disease, orchestrating this immune activation. Among IL-1 family cytokines, IL-18 has been the most thoroughly investigated in SLE. Monocytes/macrophages and dendritic cells are the major source of IL-18, produced as precursor protein and then enzymatically cleaved to give rise to the mature active cytokine [1]. IL-18 activity is regulated by IL-18 binding protein (IL-18BP), a soluble molecule that binds mature IL-18 with high affinity and prevents its interaction with cell surface receptors [2]. The interaction of IL-18 with IL-18BP is a stoichiometric 1:1 interaction with a K_d_ of 400 pM. Thus, the level of free IL-18, which is able to interact with the membrane receptors and thus is biologically active, depends on the absolute concentrations of both IL-18 and IL-18BP, and on their affinity of interaction, according to the law of mass action. IL-18 indirectly increases the production of its own inhibitor in a feedback loop, through up-regulation of the major IL-18BP inducer, interferon-γ (IFN-γ) [3]. In pathological conditions characterised by high levels of IL-18, IL-18BP levels are also increased, in the attempt to counteract the inflammatory effects of IL-18.

Measurable concentrations of IL-18 are detectable in serum from normal subjects, whereas in normal conditions the highly inflammatory cytokine IL-1β is virtually absent in the circulation. The activity of IL-1β and of the closely related IL-1α is finely tuned by the differential expression of a group of receptors and soluble inhibitors. IL-1R1 is the signalling receptor, which binds IL-1 and recruits the accessory chain IL-1R3 for initiating signalling, while the IL-1-like soluble antagonist IL-1Ra binds to IL-1R1, thereby preventing binding of agonist IL-1 molecules, and does not allow association of IL-1R3, thus inhibiting signal transduction. The soluble form of IL-1R1 acts as a decoy inhibitor, by capturing IL-1 cytokines in solution, whereas the soluble form of IL-1R3 may form complexes with IL-1 bound to soluble receptors. Both in its membrane form and as soluble protein, IL-1R2 binds IL-1β with high affinity and recruits IL-1R3, but is unable to activate intracellular signalling, behaving as a decoy receptor [4]. The tight regulation of IL-1 activity underlines its potent inflammatory activity, which needs control to avoid pathological derangements. Indeed, excessive IL-1 activity is at the basis of a wide range of diseases (chronic inflammatory autoimmune, degenerative, cardiovascular, autoinflammatory, etc.), as suggested by the ameliorative effects of anakinra (the recombinant IL-1Ra drug) and canakinumab (the anti-IL-1β antibody) [5–7].

IL-33 is another member of the IL-1 family, highly homologous to IL-18, which contains a nuclear localisation signal and acts as a transcription regulating factor. IL-33 can be released extracellularly as full-length protein through cell death pathways and it can be cleaved to shorter forms with increased cytokine activity. Acting as a cytokine and binding to its receptor IL-1R4 (previously known as T1/ST2), IL-33 drives type 2-dependent inflammation and also tissue repair, and it is involved in allergic and lung/mucosal inflammation [8–10]. The soluble form of the receptor, sST2/sIL-1R4, is a natural inhibitor of IL-33.

A large body of evidence from animal models and human disease supports the role of IL-18 in SLE. In the MRL *lpr*/*lpr* murine lupus model, up-regulation of IL-18 expression is detected in all affected organs, including nephritic kidneys [11–13]. Intraperitoneal administration of recombinant IL-18 has been demonstrated to exacerbate renal disease [11] and in vivo inhibition of IL-18 by anti-IL-18 cDNA vaccination attenuates lymphoproliferation and nephritis and increases lifespan [14].

In human disease, increased glomerular expression of IL-18 is found in kidney biopsies from patients with SLE [15, 16], and local production of the cytokine is reported to play an important role in driving the migration of dendritic cells to the kidney [16]. High levels of IL-18 and IL-18BP have been observed in patients’ serum; despite the overproduction of IL-18BP, free IL-18 levels are increased, especially in the patients with active disease [17, 18].

At variance with IL-18, the role of other inflammatory cytokines of the IL-1 family, and of their regulation by soluble receptors, is much less known. The aim of the present work was to identify possible correlation between levels of cytokines/receptors and the clinical and serological features of SLE, by performing a comprehensive analysis of IL-1 family cytokines and receptors in serum from patients with SLE.

## Methods

### Patients

Seventy-four patients attending the Clinical Immunology and Rheumatology Units of the University of Pisa, who satisfied the revised American College of Rheumatology (ACR) criteria for the diagnosis of SLE, were consecutively recruited in this study [19]. In all patients, a full clinical and serological evaluation was performed that included measuring the levels of C3 and C4, anti-dsDNA and anti-C1q antibodies. Anti-dsDNA antibodies were detected by immunofluorescence on *Crithidia luciliae* (CLIF test) and by a solid phase immunoenzymatic assay employing genomic DNA, as previously described [20]. Anti-C1q antibodies were detected by ELISA as previously described [21].

On the basis of clinical and serological findings, a disease activity score (the European Consensus Lupus Activity Measurement (ECLAM) index) was calculated; a score higher than or equal to 2.5 was considered indicative of active disease. Eighty sex-matched and age-matched blood donors formed the control group.

### Cytokine detection

Cytokines of the IL-1 family (IL-1α, IL-1β, IL-33, IL-18), soluble receptors (sIL-1R1, sIL-1R2, sIL-1R3, sIL-1R4) and antagonists (IL-1Ra, IL-18BP) were measured in serum by a custom-made multiarray ELISA (Quansys Biosciences, Logan, UT, USA). Free IL-18 was calculated as the amount of IL-18 not inhibited by IL-18BP [22]. Briefly, by knowing the molecular weight of both IL-18 (18.4 kDa) and IL-18BP (17.6 kDa), that IL-18 and IL-18BP interact at a ratio of 1:1, and that this interaction has a K_d_ of 0.4 nM, the law of mass action:

$$ \mathrm{Kd}=\frac{\left[\mathrm{Ligand}\right]\cdotp \left[\mathrm{Receptor}\right]}{\left[\mathrm{Ligand}\cdotp \mathrm{Receptor}\right]} $$

has been applied as follows:

$$ x=\frac{-b+\sqrt{b^2-4c}}{2} $$

where *x* is [IL-18]_free_, *b* is [IL-18BP] – [IL-18] + K_d_, and *c* is – K_d_ • [IL-18].

Informed consent according to the Declaration of Helsinki was obtained from all the subjects and the study was approved by the local Ethics Committee (protocol 3661/2012).

### Statistical analysis

Statistical analysis was performed using IBM-SPSS Statistics (version 20), and XLXSTAT software, for Mac. After the application of a test for normality, values were expressed as mean and standard deviation if the distribution was Gaussian and as median and interquartile range otherwise.

The Mann-Whitney test or, if appropriate, the unpaired *t* test was used to determine differences between patients and controls or between patients with active (ECLAM *≥* 2.5) and inactive disease. Association was evaluated by Spearman correlation testing.

Data were then analysed to identify the serological markers maximally contributing to group separation of patients based on the following outcome variables: (1) disease activity, (2) presence of active nephritis, (3) haematological involvement. To this end, three partial least square (PLS) models were generated (one for each outcome variable), and the markers with variable importance in projection (VIP), expressing a measure of a variable’s relevance in the model) greater than 1.25 were considered significant for separation of the set. Briefly, PLS has similarities to principal component analysis (PCA), a technique able to extract the relevant information from the data table, and to represent it as a set of new orthogonal variables called principal components. The major difference is that with PCA, the principal components are determined solely using the data values of the *X* input variables (in this case serological markers), whereas with PLS the data values of both the *X* and the *Y* outcome variables (*Y*_1_: disease activity or not, *Y*_2_: presence of nephritis or not, *Y*_3_: haematological involvement or not, respectively) influence the construction of components, improving the predictive value of the input variables [23]. *P* values <0.050 were considered significant in all statistical analyses.

## Results

### Patient cohort

Seventy-four patients with SLE were included in this study. Demographic and clinical characteristics are given in Table 1. Twenty-seven patients were classified as having active disease (ECLAM ≥2.5).

**Table 1:** Demographic and clinical features of patients with systemic lupus

Demographic features		Value
Female/male ratio		64/10
Age at study (years), median (IQR)		39.5 (30–50)
Disease duration (years), median (IQR)		10 (4–20)
Disease activity (ECLAM), median (IQR)		1.75 (0–3.1)
Clinical manifestations, percentage		
Arthralgia/arthritis		44%
Haematological manifestations		34%
Skin manifestations		23%
Active nephritis		23%
Nervous system involvement		9%
Serositis		5%
Serological features, percentage		
Complement	Low C3 levels	63%
	Low C4 levels	26%
Anti-DNA Ab	CLIF test	46%
	ELISA	49%
Anti-complement C1q Ab		52%

*ECLAM* European Consensus Lupus Activity Measurement, *Ab* antibodies

All except for 3 of the patients were treated with steroids, 55 patients at a dosage lower than 10-mg-equivalent and 16 patients at a dosage between 10 and 50-mg-equivalent prednisolone. Steroid therapy was started at disease onset. At the time of blood sampling, 46% of patients were treated with hydroxychloroquine, 36% with mycophenolate mofetil, 11% with cyclosporine and 3% with methotrexate. In the patients with active SLE, blood samples were obtained prior to the use of high dose steroids or cyclophosphamide.

On the basis of clinical and serological features, patients were divided into subsets according to organ involvement at serum sampling. Joint involvement (arthralgia and/or arthritis) was detected in 44% of the patients; haematological involvement (haemolytic anaemia and/or leukopenia and/or thrombocytopenia) was detected in 34%, and active nephritis in 23% of patients. In the 17 patients with active renal disease, creatinine levels were between 0.63 and 1.63 mg/dl; urinary proteins were between 0.4 and 8.8 g/24 h and serum albumin was between 2.4 and 3.6 g/dl.

### IL-1 family cytokines and receptors in SLE

**Fig. 1:**
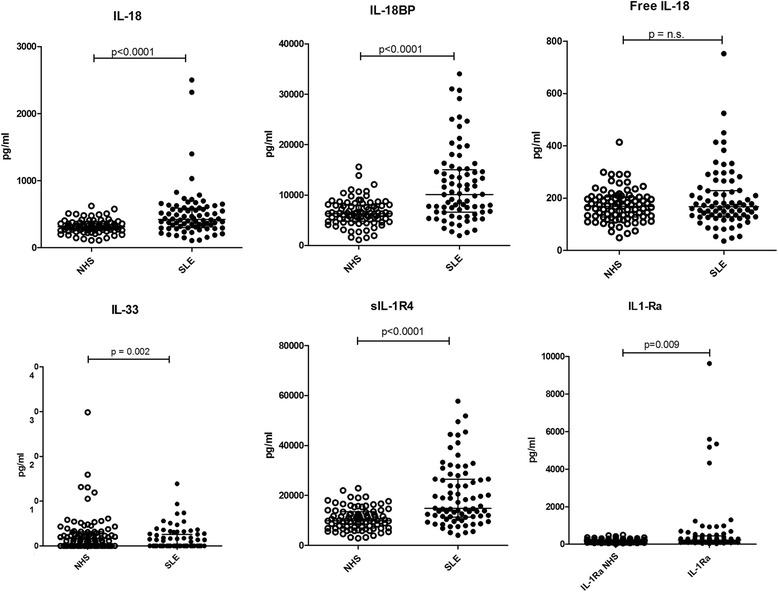
Levels of soluble cytokines and receptors of the IL-1 family in patients with systemic lupus erythematosus (SLE) vs. normal healthy controls. IL-18, IL-18 binding protein (IL-18BP), free IL-18, IL-33, soluble IL-1 receptor 4 (sIL-1R4) and IL-1 receptor antagonist (IL-1Ra) were measured in serum from 80 normal healthy subjects (NHS) and 74 patients with SLE. Data are expressed as pg/ml of serum in patients with SLE compared to controls, by means of the non-parametric Mann-Whitney test. Horizontal bars represent medians and IQR; *p* values are given in each panel

Total IL-18 and IL-18BP were both significantly higher in patients with SLE (*p* < 0.0001) compared to cytokine levels in normal controls, whereas free IL-18 was not significantly different (Fig. 1, Table 2).

**Table 2:** Cytokine and receptor levels in normal controls (NHS) and patients with systemic lupus (SLE) expressed as pg/ml of serum

Cytokine/receptor	NHS	SLE	*p*
	Median (IQR)	Median (IQR)	
IL-1α	0 (0–0)	0 (0–0)	0.656
IL-1β	0 (0–0)	0 (0–0)	0.951
IL-1Ra	178.2 (123.8–229.1)	219.8 (127.5–436.8)	0.0094
sIL-1R1	1143 (1014–1247)	1115 (949.5–1378)	0.629
sIL-1R2	4515 (3008–5727)	3841 (2093–5525)	0.251
sIL-1R3	18886 (16660–22581)	20384 (16971–23109)	0.260
sIL-1R4	9894 (6917–13515)	14782 (11325–26521)	< 0.0001
IL-33	1.7 (0–3.2)	0 (0–2.55)	< 0.002
IL-18	313.3 (261.9–359.3)	421.0 (299.7–592.1)	< 0.0001
IL-18BP	6302 (4808–8014)	10121 (6630–15033)	<0.0001
Free IL-18	168.8 (127.7–204.2)	167.2 (129.4–228.8)	0.526

*IL-1Ra* IL-1 receptor antagonist, *sILR1-–4* soluble IL receptor 1−4, *IL-18BP* IL-18 binding protein,

IL-33 was significantly lower in SLE (*p* = 0.002) and sIL-1R4, its natural inhibitor, significantly higher (*p* < 0.0001) (Fig. 1, Table 2). Similarly, IL-1Ra levels were higher in patients with SLE than in controls (*p* = 0.009). No significant differences were detected in the levels of IL-1α, IL-1β, sIL-1R1, sIL-1R2 or sIL-1R3 (Table 2).

### Cytokines, complement and autoantibodies

On analysing the relationships between cytokines levels, complement components and titres of autoantibodies, we found that sIL-R4 levels were inversely correlated with C3 levels (*R* = -0.301, *p* = 0.01), and positively correlated with anti-C1q and anti-dsDNA antibody levels (*R* = 0.267, *p* = 0.03 and *R* = 0.418, *p* = 0.0005, respectively). sIL-1R2 levels were positively correlated with C3 levels (*R* = 0.375, *p* = 0.001) and negatively correlated with anti-C1q antibodies (*R* = -0.303, *p* = 0.02). Similarly, total IL-18 and free IL-18 were positively correlated with anti-C1q antibodies (*R* = 0.323, *p* = 0.01 and *R* = 0.277, *p* = 0.03, respectively), and anti-dsDNA antibodies levels (*R* = 0.292, *p* = 0.02 and *R* = 0.371, *p* = 0.002, respectively).

### Cytokines and disease activity

**Fig. 2:**
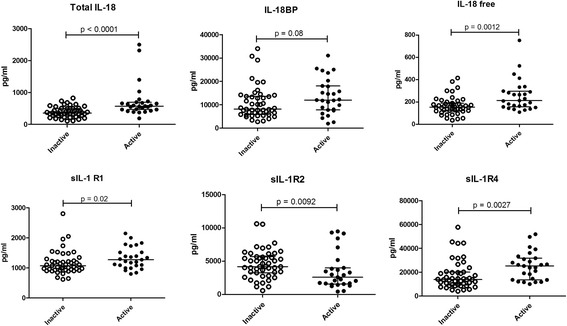
Levels of soluble cytokines and receptors of the IL-1 family in patients with inactive and active systemic lupus erythematosus (SLE). IL-18, IL-18 binding protein (IL-18BP), free IL-18, soluble IL-receptor 1 (sIL-1R1), sIL-1R2 and sIL-1R4 were measured in serum from 40 patients with inactive SLE and 22 patients with active SLE. Data are expressed as pg/ml of in patients with inactive SLE compared to patients with active SLE, by means of the non-parametric Mann-Whitney test. Horizontal bars represent medians and IQR; *p* values are given in each panel

In patients with active SLE, we detected significantly higher IL-18 (*p* < 0.0001) and free IL-18 (*p* = 0.0012), as compared with patients with inactive SLE (Fig. 2). sIL-1R4 and sIL-1R1 were higher in patients with active disease (*p* = 0.0027 and *p* = 0.02, respectively), while higher sIL-1R2 was detected in patients with inactive disease (*p* = 0.0092) (Fig. 2).

To further explore the relationship between the levels of IL-1 family cytokines and receptors and disease activity, correlation with the disease activity index was tested. IL-18, free IL-18 and sIL-1R4 were all directly correlated with the ECLAM (*R* = 0.351, *p* = 0.002; *R* = 0.238, *p* = 0.04 and *R* = 0.383, *p* = 0.0008, respectively). sIL-1R2 was inversely correlated with the ECLAM (*R* = -0.251, *p* = 0.03).

In the subgroup of patients with active renal disease, there was no correlation between proteinuria and IL-1α, IL-1β, IL-33, total and free IL-18, sIL-1R2, sIL-1R3 or sIL-1Ra. On the contrary, there was positive correlation between sIL-1R4 and proteinuria (*R* = 0.66, *p* = 0.0038) and also between sIL-1R1 and proteinuria (*R* = 0.52, *p* = 0.034).

### Multivariate data analysis

Three partial least square (PLS) models were generated to identify the cytokines maximally contributing to group separation of patients according to disease activity, presence of active nephritis or haematological involvement. Using the ECLAM as the *Y* outcome variable to distinguish between patients with active and inactive disease, PLS identified sIL-1R4 (VIP = 2.440), sIL-1R2 (VIP = 1.29) and anti-dsDNA (VIP = 1.253). Higher sIL-1R4 (25142 ± 11571 vs. 17676 ± 11930; *p* < 0.01) and anti-dsDNA (335 ± 518 vs. 42 ± 125; *p* < 0.01), and lower sIL-1R2 (3509 ± 2768 vs. 4633 ± 2137; *p* < 0.01) were detected in patients with active disease compared with patients with inactive disease.

**Fig. 3:**
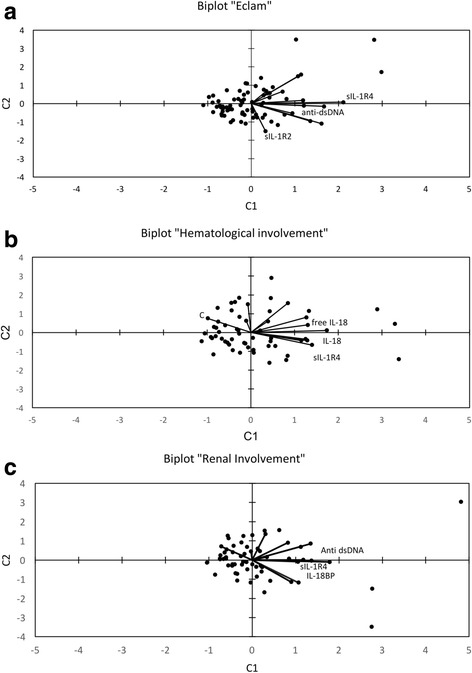
Partial least squares (PLS) analysis. PLS biplots relating serological markers to (i) the European Consensus Lupus Activity Measurement (ECLAM) score (**a**), (ii) renal involvement (**b**) and (iii) haematological involvement (**c**). The vectors and the dots show the serological markers and the observations, respectively. Labels on vectors indicate input variables with a variable importance in protection (VIP) greater than 1.25, i.e., those variables relevant in discriminating between patients with active and inactive disease (**a**), patients with and without nephritis (**b**) and patients with and without haematological involvement (**c**). The near zero angle between soluble IL-1 receptor 4 (sIL-1R4) and anti-dsDNA (**b**), and among sIL-1R4, IL-18 and free IL-18 (**c**) shows the association between these variables. IL-18BP, IL-18 binding protein

Figure 3a shows a biplot of the PLS analysis, with sIL-1R4 and anti-dsDNA on the top right and sIL-1R2 on the bottom right, respectively. The opposite direction of sIL-1R2 with respect to sIL-1R4 and anti-dsDNA underlines that this cytokine is lower in the subjects with active disease.

Variables identifying patients with active nephritis were sIL-1R4 (VIP = 1.849), IL-18BP (VIP = 1.605), and anti-dsDNA (VIP = 1.524). Comparing subjects with or without active renal involvement, higher values of sIL-1R4 (27217 ± 13035 vs. 17660 ± 9911, *p* < 0.01), of IL-18BP (11140 ± 11202 vs. 5045 ± 6334; not significant) and of anti-dsDNA (360 ± 546 vs*.* 46 ± 122; *p* < 0.01) were observed in active nephritis. The biplot in Fig. 3b shows that sIL-1R4, anti-dsDNA and IL-18BP, with spatially close vectors, represent the markers mainly contributing to the separation of the set (renal involvement vs. no involvement).

The relevant markers of haematological involvement appeared to be sIL-1R4 (VIP = 1.691), C3 (VIP = 1.644), IL-18 (VIP = 1.311) and free IL-18 (VIP = 1.267). In fact, in discriminating between patients with and without haematological involvement, higher sIL-1R4 (24649 ± 12436 vs. 17279 ± 9992, *p* < 0.05), IL-18 (696 ± 591 vs. 396 ± 154, *p* < 0.01), free IL-18 (257 ± 161 vs. 166 ± 72, *p* < 0.05) and lower C3 (59 ± 25 vs. 91 ± 30, *p* < 0.001) were detected. The adjacent vectors in Fig. 3c represent a relationship between sIL-1R4, IL-18 and free IL-18. Furthermore, the opposite direction of C3 (top left) with respect to sIL-1R4, IL-18, free IL-18 (bottom right) underlines that this factor is lower in the individuals with haematological complications.

## Discussion

In this paper, we analysed the serum levels of cytokines and receptors of the IL-1 family in a cohort of patients with SLE. The data obtained suggest an important role of several IL-1 family members as biomarkers of disease activity or organ involvement. The association of single variables (cytokines, receptors, complement and anti-dsDNA levels) with disease activity and disease manifestations was explored. Moreover, clusters of variables were identified that characterise patients with active disease or patients with defined clinical features.

Among IL-1 family cytokines, we confirmed the relevance of serum IL-18 in discriminating patients with SLE from normal controls. As previously observed [17, 18], IL-18BP is also increased in patients with SLE, but despite this increase, free IL-18 is still higher in patients with active SLE. Higher free IL-18, that is, the cytokine able to bind its receptor and exert all its activities, is in fact a feature of patients with active disease and of patients with haematological involvement.

The correlation between IL-18 or free IL-18 and other well-known biomarkers of active disease, such as anti-C1q and anti-dsDNA antibodies, suggests an important role of IL-18 in the pathogenesis of the disease and also in mediating inflammation in the active stages. Additional evidence for the critical role of IL-18 is provided by multivariate analysis, which identified lower IL-18BP and higher sIL-1R4 as distinguishing features of patients with active nephritis. A decrease in the dampening of IL-18-mediated inflammation at the local level may thus contribute to active renal disease. Previous studies, including follow up studies similarly proposed a role for IL-18 both in inducing the immune system abnormalities typical of SLE and in triggering disease exacerbations [17, 18, 24, 25].

At variance with the previously described associations with active disease and nephritis, the relationship between free IL-18 levels and haematological manifestations represents a novel finding. Higher free IL-18 levels, together with high sIL-1R4 and low C3 in fact, characterise patients with haematological involvement. In idiopathic thrombocytopenic purpura, a haematological disease that in some cases heralds systemic lupus, elevated serum IL-18 and higher expression of IL-18 mRNA in peripheral blood mononuclear cells (PBMC) were detected [26]. Even if free IL-18 was not calculated, the ratio of IL-18 and IL-18BP was higher in active disease and normal in remission, suggesting defective suppression of IL-18 signalling, similar to what is observed in SLE.

In contrast with IL-18, no increase in IL-1α or β was detected and, among soluble IL-1 receptors and inhibitors, only IL-1Ra was higher in patients vs. controls. Higher production of IL-1Ra by SLE monocytes has been previously reported [27, 28] and recently confirmed by mass cytometry analysis [29]. Previous data on the correlation between IL-1Ra levels and disease activity are conflicting [27, 28, 30], and the role of IL-1Ra may differ according to organ involvement. In fact, no increase of IL-1Ra in active nephritis and higher levels of IL-1Ra in extra-renal flares have been observed [31]. In our group of patients, there was no correlation between IL-1Ra and disease activity, complement or autoantibody levels or organ involvement. These data suggest that, at variance with other inflammatory disorders like rheumatoid arthritis, IL-1Ra may have limited involvement in the regulation of IL-1 activation in SLE. The two regulators of IL-1 that correlate with disease activity in this study are sIL-1R1 and sIL-1R2. Both these molecules act as decoy receptors for IL-1. However, the two soluble receptors differ substantially in their capacity to inhibit IL-1. In fact, sIL-1R1 can also bind IL-1Ra with high affinity, therefore partially losing its IL-1 inhibitory capacity and becoming an inhibitor of the inhibitor [32]. On the other hand, sIL-1R2, present in serum at high concentrations and endowed with a specific binding capacity for IL-1, is the main inhibitor of IL-1 [32]. In patients with SLE, sIL-1R1 is increased in active disease and positively related with ECLAM, while lower levels of sIL-1R2 are detected in patients with active disease and, together with sIL-1R4, defines the subset of patients in the active stage of the disease. Thus, the increase in sIL-1R1 in active disease may contribute to dampening the IL-1Ra-mediated protective effects, while the decrease in sIL-1R2 confirms the involvement of IL-1-mediated inflammation in active disease and the protective role of this soluble decoy receptor.

This opposite trend of the two receptors levels suggests the involvement of different proteases in the shedding of IL-1 receptors from the membrane. It has been reported that ADAM17 can release IL-1R2 but not IL-1R1 [33], and higher mRNA levels of this disintegrase are detectable in PBMC from patients with SLE [34], but no data are so far available on protein levels or enzymatic activity. While sIL-1R1 is released from the membrane upon cleavage by a metalloprotease [35, 36], its ligand-free form is likely generated by alternative splicing [37], opening another level of possible disease-related regulation.

In patients with SLE, serum levels of IL-33 were lower than in normal controls. IL-33 signalling is mediated by the binding to ST2/IL-1R4 and subsequent recruitment of the accessory chain IL-1R3. The soluble form of the receptor, generated by alternative splicing, can bind IL-33, preventing its interaction with membrane IL-1R4, and thus behaving as a soluble decoy receptor. The levels of sIL-1R4 are increased in several inflammatory disorders [32, 38, 39]. In our cohort of patients with SLE, sIL-1R4 was increased, especially in the patients with active disease, and was highly correlated with the disease activity index and with anti-dsDNA and anti-C1q antibody levels. In the subgroup of patients with active nephritis, sIL-1R4 is correlated with urinary proteins. Moreover, multivariate analysis identified sIL-1R4 as the most relevant variable in discriminating patients with active and inactive disease, with sIL-1R2 and anti-dsDNA contributing to a lesser extent. In the identification of patients suffering from active nephritis, sIL-1R4 contributes to a similar extent as anti-dsDNA and IL-18BP. Together with IL-18 and reduced C3, sIL-1R4 identifies patients with haematological involvement. Thus, sIL-1R4 emerged as the most interesting biomarker among the soluble receptors of the IL-1R family.

Mok et al. [40] similarly reported higher levels of sIL-1R4 in patients with SLE, which correlated with levels of anti-dsDNA antibodies and with the Systemic Lupus Erythematosus Disease Activity Index (SLEDAI) and correlated negatively with C3 levels. Linear regression analysis showed that sIL-1R4 was an independent predictor of greater disease activity, irrespective of organ involvement. At variance with that report, we identified sIL-1R4 as predictive of active disease but also of active nephritis and haematological involvement. A different SLE cohort and a different proportion of patients with active renal disease in the group of patients with active disease may explain this discrepancy. Caucasian patients with SLE were recruited in this study and Asian patients were recruited by Mok et al. [40], but on the whole the data are concordant in describing sIL-1R4 as an important biomarker in SLE across genetic differences. Correlation between sIL-1R4 levels and proteinuria has been reported by Zhang et al. [41] in IgA nephropathy, suggesting the possibility that sIL-1R4 may also be elevated in other kidney disorders characterised by heavy proteinuria.

IL-1R4 is expressed on different cell types: T helper 2 (Th2) cells, dendritic cells, M2 macrophages, mast cells, eosinophils, basophils and innate lymphoid cells (ILC) type 2. Acting on these cells, IL-33 orchestrates type 2 immunity, also promoting the reparative capacity of macrophages and ILCs and thus contributing to chronic fibrotic disorders [9, 42]. An important role of the IL-33/IL-1R4 axis has been proposed in different diseases, such as asthma, inflammatory bowel disease and rheumatoid arthritis. Increased serum sIL-1R4 has been described in these conditions [43]. However, sIL-1R4 has been thoroughly investigated as a biomarker in cardiovascular disease only. In fact, it is associated with adverse outcome in myocardial infarction, heart failure and pulmonary disease and is presently considered the strongest predictor of mortality in acute or chronic heart failure [44].

Only limited data are available on the IL-33/IL-1R4 axis in SLE. Increased serum IL-33 has been reported only in Chinese patients with SLE [45], and a role of IL-33 has been suggested in the interstitial renal fibrosis that characterises later stages of lupus nephritis [46]. In animal models of the disease, the administration of a monoclonal antibody against IL-33 to lupus-prone MRL/*lpr* mice reduced the deposition of immune complexes and the severity of nephritis, affecting the titre of anti-dsDNA antibodies and the level of inflammatory cytokines [47].

In SLE, sIL-1R4 may represent an attempt to limit the inflammatory effects of IL-33, similarly to the role of IL-18BP in controlling IL-18 signalling. In this perspective, the low IL-33 levels in a disorder characterised by an increased rate of cell death and reduced clearance of dying cells [48, 49] may represent the result of overproduction of the soluble receptor. The role of IL-33 and its receptor in SLE should be further investigated. In fact, no data on the expression of IL-1R4 in the cells and tissues of patients with SLE are available, and no study on lupus-prone mice knockout (KO) for IL-33 or IL-1R4 genes has so far been conducted. Available data, however, support the use of sIL-1R4 as a biomarker in SLE.

## Conclusions

On the whole, the present study confirms the role of “traditional” biomarkers such as anti-dsDNA and C3, strengthens previous observations of the utility of IL-18 measurement and supports the introduction of sIL-1R2 and sIL-1R4 as novel biomarkers. Moreover, the data obtained in this study suggest that IL-1 activation is tightly controlled at the tissue level in SLE, and a failure of inhibitory circuits may be critical in triggering the active stages of the disease.

## Abbreviations

ECLAM: European Consensus Lupus Activity Measurement
ELISA: Enzyme-linked immunosorbent assay
IL: Interleukin
IL-18BP: Interleukin 18 binding protein
IL-1Ra: Interleukin 1 receptor antagonist
ILC: Innate lymphoid cells
PBMC: Peripheral blood mononuclear cells
PCA: Principal component analysis
PLS: Partial least square
sIL-1R: Soluble interleukin 1 receptor
SLE: Systemic lupus erythematosus
VIP: Variable importance in projection
